# Inflammatory Aetiology of Human Myometrial Activation Tested Using Directed Graphs

**DOI:** 10.1371/journal.pcbi.0010019

**Published:** 2005-07-22

**Authors:** Andrew M Bisits, Roger Smith, Sam Mesiano, George Yeo, Kenneth Kwek, David MacIntyre, Eng C Chan

**Affiliations:** 1 Mothers and Babies Research Centre, Hunter Medical Research Institute, John Hunter Hospital, Newcastle, Australia; 2 Departments of Reproductive Biology and Ob/Gyn, Case School of Medicine, University Hospitals of Cleveland, Ohio, United States of America; 3 KK Women's and Children's Hospital, Singapore, Singapore; University of California at San Diego, United States of America

## Abstract

There are three main hypotheses for the activation of the human uterus at labour: functional progesterone withdrawal, inflammatory stimulation, and oxytocin receptor activation. To test these alternatives we have taken information and data from the literature to develop causal pathway models for the activation of human myometrium. The data provided quantitative RT-PCR results on key genes from samples taken before and during labour. Principal component analysis showed that pre-labour samples form a homogenous group compared to those during labour. We therefore modelled the alternative causal pathways in non-labouring samples using directed graphs and statistically compared the likelihood of the different models using structural equations and D-separation approaches. Using the computer program LISREL, inflammatory activation as a primary event was highly consistent with the data (*p* = 0.925), progesterone withdrawal, as a primary event, is plausible (*p* = 0.499), yet comparatively unlikely, oxytocin receptor mediated initiation is less compatible with the data (*p* = 0.091). DGraph, a software program that creates directed graphs, produced similar results (*p* *=* 0.684, *p* *=* 0.280, and *p* = 0.04, respectively). This outcome supports an inflammatory aetiology for human labour. Our results demonstrate the value of directed graphs in determining the likelihood of causal relationships in biology in situations where experiments are not possible.

## Synopsis

This paper describes how novel computational approaches have been used to test hypotheses for important physiological events when the traditional approaches of animal studies and experiment are not possible. The processes that regulate the onset of human labour are presently unknown, principally because there are no good animal models for human pregnancy and because it is unethical to conduct experiments on pregnant women undergoing labour. However, several hypotheses have been advanced to explain the trigger for labour, including: a functional withdrawal of the hormone progesterone, increased inflammation in the uterus, and increased signalling through the hormone oxytocin. To test these hypotheses the researchers used data on the messenger RNA concentrations of critical variables in samples of uterine muscle taken from 12 women undergoing caesarean section prior to labour and 12 women during labour. Directed graphs for each of the proposed hypotheses were then generated, where the graphs represent the direction of causal influence between different variables. Statistical testing determined how well the graphs of each hypothesis matched the experimental data. The results strongly support an inflammatory origin for the onset of human labour. This approach could be applied to other problems in human biology where the traditional approaches of experiments and animal models are not possible.

## Introduction

In most mammals, pregnancy is maintained by high maternal plasma concentrations of progesterone, and labour occurs when progesterone concentrations fall. However, parturition in humans is unusual, as maternal progesterone levels remain high until delivery. The mechanisms regulating human parturition remain obscure. Experimental studies to resolve this uncertainty are restricted because of ethical considerations. In this setting it is not surprising that predictors of premature birth and treatments for preterm labour are generally of limited efficacy [1]. The ignorance regarding human parturition is costly, as rates of premature birth are increasing and premature birth is a major cause of neonatal death and cerebral palsy [2]. Recent advances in causal pathway modelling using directed graphs raise the possibility of advancing knowledge in this area without the need for interventional experiments [3].

We began by gathering knowledge regarding potential variables that might play a part in a causal pathway leading to delivery in humans. Three major hypotheses have been advanced for the onset of normal parturition in humans: an endocrine pathway commencing with a functional progesterone withdrawal, an inflammatory stimulated process, and an oxytocin-mediated mechanism.

In the functional progesterone withdrawal pathway, an unknown factor stimulates expression of the A type of the progesterone receptor (PRA), which acts as a dominant negative repressor of the progesterone-signalling B type receptor (PRB) [4–6]. Functional progesterone withdrawal leads to increased expression of estrogen receptor α (ERα) and, hence, activation of contraction-associated proteins such as oxytocin receptor (OTR) [7] and the prostaglandin synthetic enzyme cyclooxygenase-2 (COX-2) [8]. Support from the literature for this pathway includes the effectiveness of the drug RU486 in initiating labour [9] and recent studies with progesterone administration resulting in a decreased risk of preterm delivery [10,11]. An alternative pathway begins with immune activation and the production of cytokines such as interleukin-8 (IL-8), prostaglandins, and other inflammatory factors such as manganese superoxide dismutase (MnSOD) [12,13]. These inflammatory factors lead to a functional progesterone withdrawal, possibly mediated by nuclear factor-kappa B (NF-κB) [8]. Support for this hypothesis is derived from (1) studies into preterm labour where infection of the genital tract has been implicated as a trigger [14], (2) the use of prostaglandins and mechanical methods (both stimulating inflammation) for the induction of labour [15], (3) the association between systemic infections in the mother in later stages of pregnancy and the onset of labour [16], and (4) evidence for physiological inflammation of the myometrium initiated by foetal alveolar proteins in late pregnancy [17]. For the third alternative, oxytocin has also been suggested as a mediator of myometrial activation, especially since the discovery of local production of oxytocin in the endometrium [18], the marked up-regulation of oxytocin receptors at labour [13], and the introduction of oxytocin receptor antagonists for the treatment of preterm labour [19].

## Results

**Figure 1:**
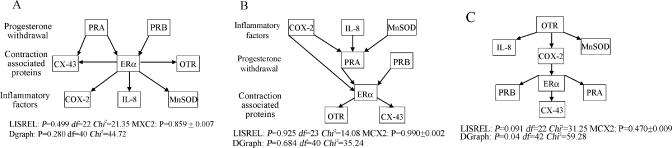
Directed Graphs of Messenger RNA Abundances in Human Myometrium Three models were generated and are represented in the following graphics. (A) Does progesterone withdrawal initiate labour? (B) Does inflammation initiate labour? (C) Does oxytocin receptor mediate onset of labour?

Using the LISREL structural equations modelling approach (with Monte Carlo analysis; Figure 1), the causal pathway modelled in the directed graph in which inflammation as represented by COX-2, IL-8, and MnSOD (*p* = 0.925; see Figure 1B) as an initiating event was almost twice as likely as the model incorporating progesterone functional withdrawal as an initiating step (*p* = 0.499; see Figure 1A), and far more likely than the model with an oxytocin receptor-mediated pathway (*p* = 0.091; see Figure 1C). The directed graph approach to assess the postulated pathways produced similar results: An inflammatory initiation pathway generated an exact *p* value of 0.684, while the progesterone withdrawal value was *p* = 0.280, and the oxytocin receptor pathway model *p* value, 0.040.

## Discussion

Our results lend support to the hypothesis that immune stimulation plays a role in the final weeks of pregnancy, eventually leading to the onset of the sustained coordinated contractions required for normal human labour. Further in vitro support for this pathway has recently been reported; in a myometrial cell line, prostaglandin PGF2α stimulated expression of PRA as predicted by the model in Figure 1B [23]. Additional strengthening of the hypothesis would come from confirmation that protein concentrations for individual inflammatory factors parallel the changes that have been observed in mRNA species. The data do not determine the aetiology of the immune activation, but such a pathway can be relatively easily extended. For example, the role of NF-κB, amniotic fluid surfactant protein A, and stretch can be tested by adding these variables to the causal pathway when data become available. Such a pathway indicates potential sites for therapeutic intervention to alter the process of labour. Labour can also be seen as a withdrawal of the factors maintaining uterine quiescence. From this perspective, inflammation can be seen as a likely factor that extinguishes uterine quiescence. It is also important to note that the cervix and other parts of the uterus may behave differently from the lower segment of the uterus from which our samples are derived. In the future, samples from these sites may provide data on additional variables to extend our knowledge of the pathways of human birth. More generally, the data illustrate the value of causal pathway modelling and directed graphs in biological situations for which experimental studies are problematic for ethical or practical reasons.

## Materials and Methods

**Figure 2:**
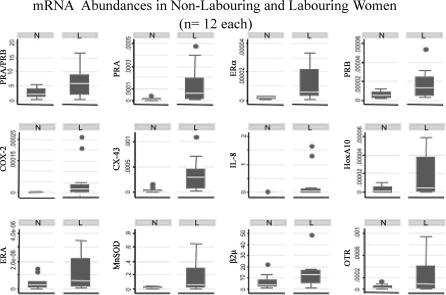
Messenger RNA Abundances in Human Myometrium Box and whisker plots of mRNA abundances of PRA and PRB, ERα, ERβ, IL-8, COX-2, MnSOD, β2-microglobulin (β2μ), connexin-43 (CX-43), OTR, and the homeobox gene HoxA10 in labouring (L) and non-labouring (N) women.

To explore the alternative hypotheses, we used data obtained from previous quantitative RT-PCR studies of relevant mRNA expression in samples of human myometrium obtained at caesarean section performed either prior to the onset of labour or during active labour (for grouped data and variables see Figure 2) [4,13]. The non-labouring samples were all taken at term, but each woman was almost certainly at different stages of a continuum leading to labour. Tissue slivers (0.5 cm × 1 cm) were obtained from the upper margins of the lower uterine segments (*n* = 12 N, *n* = 12 L). QRT-RTPCR was performed as previously described to measure the relative mRNA abundances of 11 genes that have been linked to parturition by previous studies [4,13]. QRT-RT-PCR assays used either SYBR Green (Applied Biosystems, Foster City, California, United States) as a nonspecific intercalating fluorescent dye or specific Taqman probes 5′-fluorescent labelled with either 6-FAM or VIC in a thermal cycler (ABI Prism 7700 Sequence Detector system, Applied Biosystems) linked to a Macintosh G4 (Apple Computer, Sunnyvale, California, United States).

**Figure 3:**
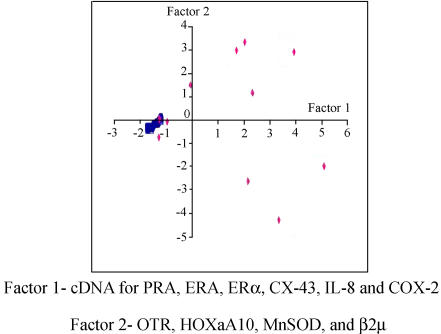
Principal Component Analysis of Messenger RNA Abundances in Human Myometrium Labouring subjects are shown by diamonds and non-labouring by squares.

Data were initially subjected to principal component analysis, which was performed using STATA (Stata Corporation, Collegeville, Texas, United States). Using the raw data, two factors were extracted that explained 61% of the total variance in the data. Factor 1, comprising cDNAs for PRA, ERα, CX-43, IL-8, and COX-2, accounted for 46% of the total variance in the data. Factor 2, comprising cDNAs for HoxA10, OTR, MnSOD, and β2μ, contributed another 15%. Each subject was scored on the basis of these factors, resulting in a graphical plot (Figure 3). Results indicated that 61% of the variance was attributable to nine variables contained in two factors, and this analysis led to a tight grouping of non-labouring samples, while labouring samples exhibited a much larger variability. The wide variability of data in the labouring tissues suggested that this condition was heterogeneous in nature. We therefore focused our pathway analysis on the more homogenous data from the non-labouring samples.

The data were transformed using normal equivalent deviates to meet the assumptions of normality while still retaining the variation of the original data (MLwiN Version 1.10.0006, Multilevel Models Project, Institute of Education, University of London, United Kingdom). Using data from the non-labouring samples, we created a directed graph for each of the literature-derived hypotheses according to the methods described by Shipley and Pearl [3,20] and based on earlier work by Wright [21] (see Figure 1).

To assess the relative likelihoods of the alternative models, we used an established program for structural equations modelling, LISREL [22], and an alternative approach, DGraph [3]. The steps for structural equations modelling are to (1) specify a causal pathway, (2) generate a series of equations that are implied by the causal network or pathway, (3) calculate parameter estimates for the equations using maximum likelihood where the objective is to choose parameter estimates that minimize the difference between observed and predicted covariance matrices, (4) calculate a variance-covariance matrix predicted by these equations and calculate a variance-covariance matrix directly from the observed data, (5) calculate the difference between these two variance-covariance matrices, and (6) calculate a probability value for the causal network based on the aforementioned difference, which follows a Chi^2^ distribution. Where there is a significant discrepancy between the observed and expected covariance matrices, the proposed causal network is unlikely. In order to deal with the relatively small sample size available for this study, using difficult-to-obtain human myometrial samples, a confidence limit for the *p*-value was calculated using Monte Carlo methods [3].

**Figure 4:**
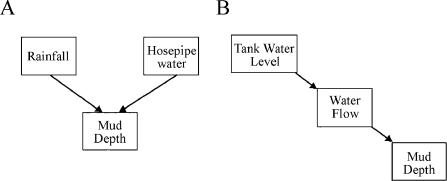
Interpreting Directed Graphs (A) Observations on rainfall, hosepipe water, and depth of mud are made. If this causal pathway is correct, the rainfall will correlate with mud depth, and hosepipe water will correlate with mud depth, but rain will not correlate with hosepipe water. However, if mud depth is fixed (also known as “conditioned”), then rainfall will correlate with hosepipe water; rainfall and hosepipe water are said to be conditionally dependent. (B) Observations are made on the level of water in a tank, water flow in a hosepipe, and depth of mud. Each of these three variables will be correlated. If, however, water flow is fixed, tank water and depth of mud will no longer be correlated. Each step of a directed graph can be statistically tested in these ways and either accepted or rejected.

The second method involves the use of directed graphs and more straightforward calculations. It is also more appropriate for small sample sizes [3]. The idea of directed graphs evolved from work in artificial intelligence. The first step in this process of inference is to formally specify a causal network, known as a directed graph, and shown in Figure 1. The rationale for the term is clear, since an explicit direction of influence is proposed. The second step is to formally acknowledge the causal implications of this graph with a series of independence statements termed “D-separation statements” (Figure 1). Central to the understanding of such causal networks is the concept of conditional independence (Figure 4), i.e., that two variables connected by a third variable, through which the path of influence is mediated A→B→C (Figure 4B), will be independent if the value of the variable B is held constant. The independence statement directly implies the causal path. In an alternative situation A→B←C (Figure 4A), A and C will be independent but will become related if B is held constant. As there are eight variables in each of our proposed networks, there are 8! possible arrangements or independence statements. Because of redundancies, a smaller number of independence statements can specify the entire causal structure. This finite set of independence statements is termed the basis set [3,20].

The third step is to statistically test the conditional independence statements listed by regressing A on B and C on B, and the residuals generated from these two equations are checked for independence using Pearson correlation or nonparametric tests, depending on the nature and distribution of the data. Since we transformed the raw data into normal equivalent deviates, Pearson correlation was appropriate, producing an exact *p* value (see Table 1).

**Table 1:**
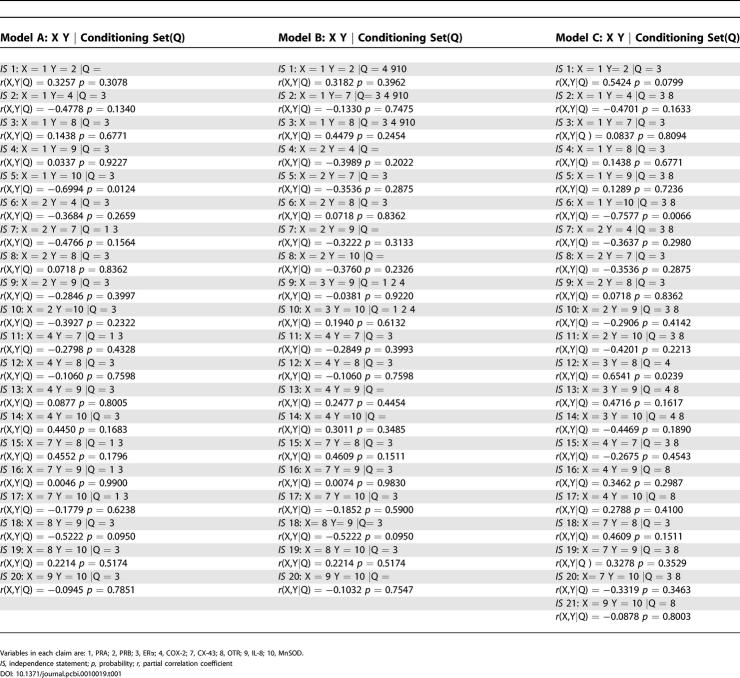
D-Separation Claims and Associated Partial Correlation Coefficients and Corresponding Probability Values

Variables in each claim are: 1, PRA; 2, PRB; 3, ERα; 4, COX-2; 7, CX-43; 8, OTR; 9, IL-8; 10, MnSOD.

*IS,* independence statement; *p,* probability; *r,* partial correlation coefficient

Finally, the overall plausibility of the model is assessed using a Fisher's C statistic [3]


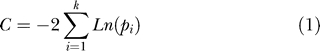


This follows a Chi^2^ distribution with 2*k* degrees of freedom, *k* being the number of independence statements.

## Supporting Information

### Accession Numbers

The Swiss-Prot (http://www.ebi.ac.uk/swissprot) accession numbers for the proteins discussed in this paper are β2μ (P61769), COX-2 (P35354), CX-43 (P17302), ERα (P03372), ERβ (Q92731), HoxA10 (P31260), IL-8 (P10145), MnSOD (Q6LEN1), OTR (P30559), PRA (P06401), and PRB (P06401).

## Abbreviations

β2μ: β2-microglobulin
COX-2: cyclooxygenase-2
CX-43: connexin-43
ER: estrogen receptor
IL-8: interleukin-8
MnSOD: manganese superoxide dismutase
OTR: oxytocin receptor
PRA: A type progesterone receptor
PRB: B type progesterone receptor
